# Use of an adaptive sensory environment in patients with autism spectrum disorder (ASD) in the perioperative environment: a parallel, randomized controlled trial

**DOI:** 10.1016/j.lana.2024.100736

**Published:** 2024-04-18

**Authors:** Sean Antosh, Chelsea Drennan, Adrienne Stolfi, Robin Lawson, Elise Huntley, Reaundra McCullough-Roach, Madelyn Hill, Tahira Adelekan, Shobhan Vachhrajani

**Affiliations:** a Department of Anesthesia, Dayton Children's Hospital, One Children's Plaza, Dayton, OH, 45404, USA; b Department of Pediatrics, Boonshoft School of Medicine, Wright State University, Dayton Children's Hospital, One Children's Plaza, Dayton, OH, 45404, USA; c Department of Surgery, Dayton Children's Hospital, One Children's Plaza, Dayton, OH, 45404, USA; d Department of Child Life, Dayton Children's Hospital, One Children's Plaza, Dayton, OH, 45404, USA; e Boonshoft School of Medicine, Wright State University, 3640 Colonel Glenn Hwy., Dayton, OH, 45435, USA; f Division of Developmental Pediatrics, Dayton Children's Hospital, One Children's Plaza, Dayton, OH, 45404, USA; g Department of Neurosurgery, Dayton Children's Hospital, One Children's Plaza, Dayton, OH, 45404, USA

**Keywords:** Autism spectrum disorder (ASD), Perioperative anxiety, Adaptive sensory environment, Randomized controlled trial (RCT)

## Abstract

**Background:**

Patients with autism spectrum disorders (ASD) experience higher rates of perioperative anxiety and are likely to receive premedication. Little is known about nonpharmaceutical interventions which may decrease anxiety. This study aims to evaluate the use of an adaptive sensory environment (ASE) to reduce ASD patient anxiety during the perioperative process.

**Methods:**

Our feasibility study (ClinicalTrials.govNCT04994613) enrolled 60 patients in two parallel groups randomized to a control (no ASE) or intervention group (ASE). We included all surgical patients aged three to twelve years, with a formal diagnosis of ASD, Asperger's Syndrome, or pervasive developmental disorder not otherwise specified. Preoperative behaviors were recorded by an unblinded nurse utilizing the validated Modified Yale Preoperative Anxiety Scale (mYPAS). The difference in score on the mYPAS was the primary outcome, and an intention-to-treat analysis was employed. A generalized estimating equations model was used to compare mYPAS scores controlling for significant independent variables.

**Findings:**

58 patients were analyzed after 1:1 randomization of 30 patients to each group. Groups were balanced except the median number of intraoperative pain medications was significantly lower in the ASE group (1 vs. 3, p = 0.012). Mean (SD) age for all patients was 7.2 (2.9) years, range 2.6–12.7. 72.4% (42/58) were White and all were Non-Hispanic or Latino. 74% were Male (21/30 ASE and 22/28 Control) and 26% were Female (9/30 ASE and 6/28 Control). No differences were found in mYPAS scores between groups at three time periods (43.5 vs. 42, p = 0.88, 47.8 vs. 48.4, p = 0.76, and 36.4 vs. 43.8, p = 0.15, ASE vs. control group, respectively). The ASE group had a significant within-group decrease in mYPAS scores from nursing intake to transition (p = 0.030).

**Interpretation:**

An ASE did not significantly reduce perioperative anxiety. However, the promising results deserve further investigation.

**Funding:**

Dayton Children’s Hospital Foundation Robert C. Cohn Memorial Research Grant.

**Research in context**

**Evidence before this study**

There is scant prospective research outlining anesthesia considerations in patients with autism spectrum disorder (ASD). We searched PubMed for research articles for the 10 years prior to March 31 2021, using the terms “general anesthesia and autism”. Three retrospective studies retrieved showed that children with ASD are known to experience higher rates of perioperative anxiety which may require pharmaceutical intervention. Often, the premedication type and route of administration is different in patients with ASD compared to those with who are neurotypical. Additionally, retrospective data review has shown adverse events are more likely to occur if a child's routines, special interest, sensory sensitivities, and level of understanding are not considered; a number of retrieved studies, including two randomized controlled trials, had examined these phenomena in the dental and radiology environments. Only one published study examined differences in perioperative anxiety between children with ASD and neurotypical children. Notably this paper did not consider nonpharmaceutical interventions which may decrease anxiety in the perioperative environment, and we were unable to find any literature to study the benefit of nonpharmaceutical interventions in a broader hospital setting and particularly in the perioperative realm.

**Added value of this study**

This is the first RCT studying the impact of an adaptive sensory environment (ASE) on perioperative anxiety in children with ASD. There was no statistically significant difference in perioperative anxiety scores, as measured on the modified Yale Preoperative Anxiety Scale, between a standard room and an ASE. Significantly less intraoperative pain medications were used in the ASE group, and there was a statistically significant reduction in anxiety score in the ASE group between the time of nursing intake and transition to the operating room, even when controlling for preoperative medications and degree of sensory sensitivity. These findings are promising for the future use of a perioperative ASE in this population, particularly as it corroborates the use of similar nonpharmaceutical interventions already seen to be beneficial in other environments.

**Implications of all the available evidence**

Previous literature has demonstrated the use of ASE and other environmental modifications to be useful in the reduction of perioperative anxiety in the dental and radiology environments. Our study corroborates this evidence, and demonstrates that such an adaptive sensory environment based on a patient's individualized coping plan can be extended to the perioperative hospital environment with all its intricacies and variability, and be applied to patients even with high sensory sensitivities as seen in this study. Future research will need to account for ASD severity characteristics and their relationship to perioperative anxiety, consider the best outcome measure for the assessment of perioperative anxiety, and should investigate the most effective specific components of an ASE and the optimal way to implement these into clinical practice, potentially in other populations that demonstrate high levels of sensory sensitivity. The use of an ASE has the potential to be infinitely beneficial in these vulnerable populations.

## Introduction

The prevalence of autism spectrum disorders (ASD) has been increasing over the last several decades and is currently estimated at 1 in 36 children in the United States. Children with ASD usually have significant language, communication, and sensory processing issues which they cope with by defining routines and rituals in their daily lives.^1^ The need for fasting in order to safely undergo anesthesia can be a difficult change to their set routine, and such changes may lead to frustration, increased anxiety, and ultimately maladaptive behaviors such as aggression to others or self-injurious behaviors.^2^ The Autistic SPACE framework summarizes these changes through the SPACE acronym comprising Sensory needs, Predictability, Acceptance, Communication, and Empathy.^3^ The SPACE authors note that every sensory modality is affected in children with ASD, and these sensitivities must be actively managed in the healthcare setting to optimize care for these children. It is well accepted that adverse events are more likely to occur if a child's routines, special interest, sensory sensitivities, and level of understanding are not considered.^4^

As the prevalence of autism spectrum disorders increases, the need for sedation and/or general anesthesia for various procedures will also increase. There is a growing body of literature that acknowledges the need for specific interventions that alleviate concerns specific to the care of children with ASD in a variety of healthcare settings. The largest contribution appears to come from the dental literature in which multiple techniques have been used, as the relationship of sensory sensitivity to difficulties in providing oral care are well established in this population.^5^, ^6^, ^7^, ^8^ Implementation of a sensory adapted dental environment (SADE), in which a number of visual, auditory, and tactile modifications were made when compared to the regular dental environment (RDE), resulted in reduction in all measures of anxiety and distress among typically developing children and those with ASD.^9^ Notably, in the Cermack et al. feasibility study, the introduction of the SADE was deemed to be feasible from practitioner and caregiver perspectives.^10^ These results were reproduced in similar studies, including in a recently published randomized trial.^11^, ^12^, ^13^ The role of interdisciplinary collaboration cannot be understated in achieving these goals.^14^^,^^15^ A recent mixed methods systematic review corroborated these findings, suggesting that the dental environment, thorough understanding of the individual autism phenotype, and the need to respond to the needs of the autistic child and caregiver are integral to providing optimal care, and a recent qualitative study of parental perceptions identified similar themes.^16^^,^^17^

Similar literature has commented on the need for modifications in the radiology environment for individuals with ASD. A Swedish expert panel survey of those involved in anesthetic delivery for radiographic procedures identified environmental features as a key aspect of managing children with ASD.^18^ Similarly, patient preparation has been identified as an integral part of successful X-ray acquisition in this population.^19^ Such considerations have been applied to more complex image acquisition paradigms, including magnetoencephalography (MEG) in which the use of sedation, often used in imaging for children with ASD, must be avoided in order to acquire ictal and functional data. MEG-PLAN (MEG Protocol for Low-language/cognitive Ability Neuroimaging), for example, incorporates an interdisciplinary approach into completion of MEG imaging using visual, tactile, and behavioral supports.^20^ Similar preparation algorithms have shown favorable results for image acquisitions in children with ASD.^21^, ^22^, ^23^, ^24^, ^25^

In the last year at Dayton Children's Hospital (DCH), we have seen over 500 patients with ASD for surgical procedures, imaging studies, and other non-invasive procedures. Children with ASD are known to experience a higher rate of perioperative anxiety, which may require pharmaceutical intervention.^26^ Typically, the premedication type and route of administration is different in patients with ASD compared to those who are neurotypical.^27^ For ASD patients, intramuscular ketamine injection may require physical restraint of the patient by their caretakers and staff leading to a potential safety risk. Currently, there is little known about nonpharmaceutical interventions which may decrease anxiety in this patient population. A survey of staff and parents of children undergoing ear, nose, and throat procedures suggested distractions, visual aids, social stories, quieter environments, separate rooms for admission, and more accurate waiting times as some strategies to improve the healthcare experience for children with ASD, however specific impacts on anxiety were not addressed.^28^ The need for individualization of care and environmental modifications is a common theme for perioperative management, but rigorous study of such interventions is limited.^4^^,^^29^, ^30^, ^31^ At some institutions clinical pathways have been implemented, however there is no published literature on their efficacy.^32^ Similarly at DCH, an individualized coping plan is developed by child life specialists prior to the day of anesthesia; these have shown benefit in the perioperative management of children with ASD and guiding preoperative sedation based on ASD severity levels.^33^

Our study aims to evaluate the use of an adaptive sensory environment (ASE) to reduce anxiety in patients with ASD during the perioperative process. Also, we will evaluate caregiver experiences and satisfaction throughout care. We hypothesize that an ASE with an individualized coping plan will decrease preoperative anxiety in a child with ASD. These environments will be most important in patients with higher degrees of sensory integration severity, as determined by the Short Sensory Profile 2 (SSP-2). Families will likely have better patient experiences and higher satisfaction ratings when an ASE is used. Long-term, we aim to establish ASEs in the perioperative space as an equal or superior alternative to premedication for patients with ASD.

## Methods

### Study design and participants

**Fig. 1:**
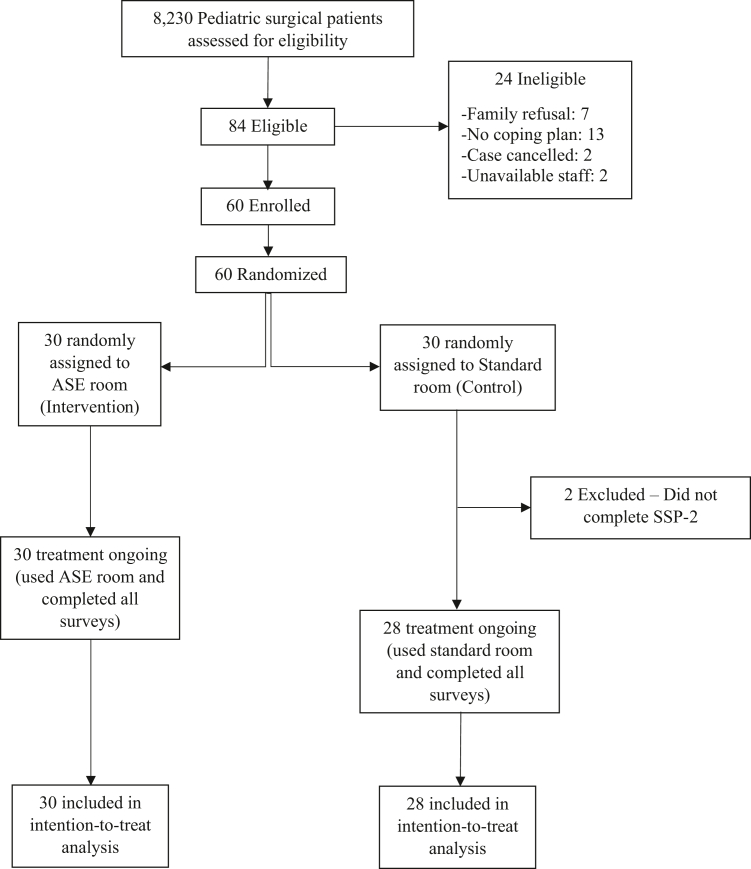
CONSORT flow diagram of participants through the trial.

We conducted a parallel, randomized clinical feasibility trial to evaluate our hypothesis that use of a perioperative ASE will reduce perioperative anxiety in patients with ASD. We recruited 60 patients at DCH in Dayton, Ohio (Fig. 1). We obtained written informed consent for all study participants and the clinical trial was approved by the DCH Institutional Review Board under DCH21-037, #1728627 (Supplementary 1). This study is registered as a Randomized Clinical Trial with ClinicalTrials.gov, number NCT04994613. Additionally, a link to the protocol on ClinicalTrials.gov is provided here.

We included all patients with a formal diagnosis of ASD, Asperger's Syndrome, or pervasive developmental disorder not otherwise specified, aged three to twelve years old, and who presented for outpatient surgery at the DCH main campus. These DSM-IV diagnostic categories were used as it reflects recording in the DCH electronic medical record. Patients were excluded if a coping plan could not be obtained prior to the day of surgery, had an American Society of Anesthesiologists (ASA) risk score greater than 3, were non-English speaking, or refused participation. Those who requested an ASE room due to prior exposure were also excluded.

A child life specialist contacted the families of surgical patients with developmental disabilities or high anxiety approximately one-week prior to surgery to develop a coping plan for the day of surgery; the coping plan details the patient's previous medical experiences and describes their communication abilities, specific interests, sensory sensitivities, best environment, comfort items, and triggers that upset the child.

The child life specialist identified those patients meeting inclusion criteria and maintained a registry of eligible patients with a completed coping plan. The coping plan was documented in the electronic health record (EHR) for review before caring for the patient on the day of surgery. The principal investigator approached eligible patients by telephone 24–48 h prior to the procedure to describe the study. Written informed consent was obtained by the research nurse or child life specialist at the time of hospital registration on the day of surgery.

### Randomization and masking

Sixty patients in two parallel groups randomized to 1:1 allocation was enrolled: 30 assigned to the control group (standard preoperative practice, no ASE) and 30 assigned to the intervention group (ASE). Patients were randomized in varying block sizes using a random number generator to ensure equal numbers in each group. Sealed numbered envelopes containing the random assignment were used, and the study nurse sequentially assigned an envelope to each enrolled patient. Allocation and randomization parameters were concealed from all study personnel until patients were allocated to a study group. Due to the nature of the intervention (ASE), blinding of study subjects or study nurse to the allocated intervention was not possible.

### Procedures

**Fig. 2:**
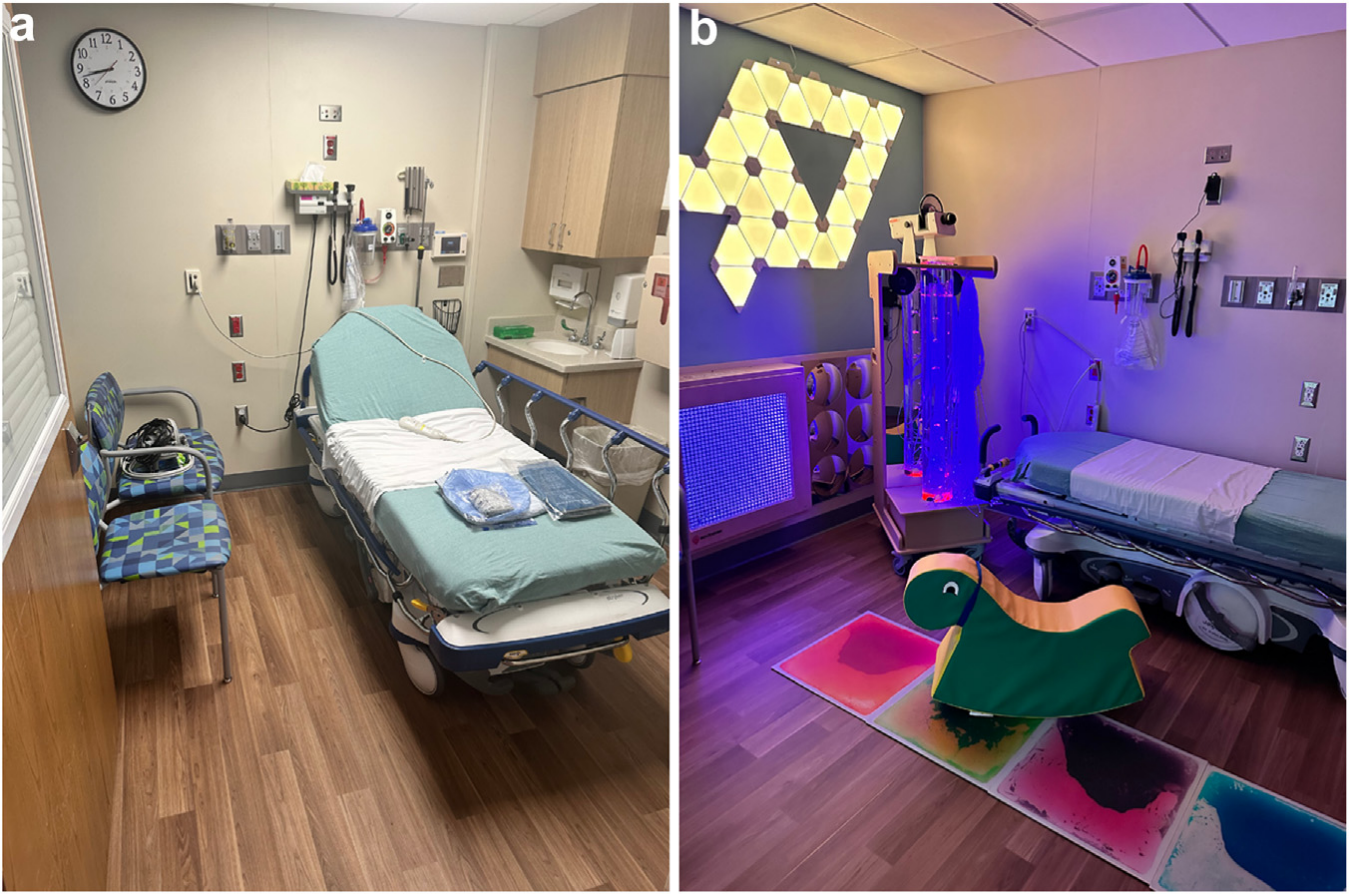
(a) Standard preoperative (control) room; (b) Adaptive Sensory Environment preoperative (intervention) room; Both images taken at Dayton Children's Hospital, Dayton, Ohio.

After registration and written informed consent was obtained, the patient was placed in the randomly allocated control or intervention room in the preoperative area. Control patients were placed in a room that did not include any additional sensory equipment as described below; these patients were allowed to use any comfort items brought from home, or were offered a hospital iPad, as is current practice for all outpatient surgery patients. Intervention group patients were placed in one of the three dedicated ASE rooms set up by nursing and child life staff according to the patient's coping plan accounting for sound, light, activity level, and other stimuli. Specific equipment included a portable popcorn tube with fiberoptic cart, handheld marble panel, color changing floor tiles, sensory fidgets (including pop its, chewies, sensory brushes, squeeze balls, and sensory chairs), and individual sensory toys (Fig. 2). Caregivers could request specific equipment to be turned off or modified but could not adjust any equipment themselves. Family requests for changes in the study group were granted so as not to cause potential anxiety or adverse harm to the patient. Time spent in each care area was also recorded, however this time was determined by individual care needs, not prespecified by intervention.

### Outcomes

Patient preoperative behaviors were recorded by the study nurse on the Modified Yale Preoperative Anxiety Scale (mYPAS) at three time points during the preoperative period: at patient registration (registration), preoperative nurse intake in the allocated room (nursing intake), and immediately prior to transition to the operating room (transition) (Supplementary 2). The primary outcome was the difference in mYPAS scores between the intervention and control groups at each time point. The mYPAS is the most widely used tool for assessing preoperative anxiety in children. It has high reliability, concurrent validity, and construct validity in children aged 2–12 years.^34^ The mYPAS consists of five items; four of the items (Activity, Emotional Expressivity, State of Apparent Arousal, and Use of Parent) are rated on scales of 1–4, and one item (Vocalizations) is rated on a scale of 1–6. The total score is determined by dividing each item rating by its highest possible rating, summing the results, dividing by 5, and then multiplying by 100. Scores on the mYPAS can range from 23.33 to 100, with higher scores representing greater anxiety. Although used in other similar studies, the mYPAS has not been validated in children with ASD.

Sensory sensitivity related to mYPAS scores, and patient satisfaction were secondary outcomes. Two questionnaires were completed by caregivers while the child was in the operating room. First, a 23-item questionnaire called Patient Experience Study Survey (PES) regarding the patient preoperative experience included evaluating discussions with the Child Life department in developing the coping plan, preoperative environment on the day of surgery, and the relationship of the perioperative team to the patient (Supplementary 3). Overall experience was rated on a 10-point Likert scale, from 1 = very dissatisfied to 10 = very satisfied.

The Short Sensory Profile 2 (SSP-2), based on Dunn's Sensory Processing Framework, was used to assess the patient's sensory processing abilities, and as a surrogate for severity of sensory sensitivity (Supplementary 4). The SSP-2 consists of 34 behavioral items measured on 5-point Likert scales from 1 = almost never to 5 = almost always. Items are divided into four quadrants for scoring, Seeking (7 items), Avoiding (9 items), Sensitivity (10 items), and Registration (8 items).^35^ Two subscale scores, Sensory Processing and Behavioral Responses Associated with Sensory Processing, as well as total scores are also determined. Raw scores are compared to a normal curve and Sensory Profile 2 Classification System developed from a normative sample. Scores >2 standard deviations (SD) below the mean are classified as “much less than others”, those between 1 and 2 SD below the mean are “less than others”, scores between 1 and 2 SD above the mean are “more than others”, and >2 SD above the mean are “much more than others”.^36^^,^^37^ The SSP-2 served as an independent variable to compare baseline responses to sensory stimuli between the control and intervention groups.

The following variables were also extracted from the EHR by study personnel blinded to randomization assignments: sex, age, weight, anesthesiologist assigned ASA and Child Induction Behavioral Assessment (CIBA), diagnosis, past medical history, procedure, home medications, preoperative medications including dose and route, intraoperative medications, event times and durations including preoperative wait time, transport to operating room time, induction to anesthesia ready, case length, and recovery time. Study participants self-reported sex assigned at birth data in the EHR and were provided with two options (male or female). All study data was originally to be housed in a password-protected Excel file, however with an IRB approved amendment before study recruitment began, all data was stored in REDCap.

### Statistical analysis

In previous randomized clinical trials using the mYPAS, investigators considered a difference between groups of ≥15 points to be clinically meaningful.^38^, ^39^, ^40^ Standard deviations reported in one of the studies ranged from 4.2–18.1.^40^ Sample size was estimated using alpha = 0.05, power = 0.80, and pooled SD = 18.1, and 23 patients were required in each group to detect a difference of 15 points in mYPAS scores between groups. Allowing for a ten percent dropout rate, 26 patients in each group, or 52 patients in total would be required, therefore we aimed to recruit 60 patients.

Univariate analyses were conducted comparing the two study groups on all independent variables, mYPAS scores (primary outcome), SSP-2 scores, PES scores, and overall satisfaction (secondary outcomes) to assess for associations. Normality was assessed with Shapiro–Wilk tests. Patient age, height, and SSP-2 scores were normally distributed and were compared between study groups with independent t tests. Patient weight and BMI, all event times in minutes, number of preoperative medications, number of intraoperative medications, and mYPAS scores were right-skewed and were compared with Mann–Whitney U tests. PES scores and overall satisfaction were left-skewed and were compared with Mann–Whitney U tests. Categorical variables were compared between groups with chi-square tests, or Fisher's exact tests if 1 or more expected cell frequencies was less than 5. The difference between the two groups in their mYPAS scores were compared at 3 time periods: registration, nursing intake, and transition. A post-hoc generalized estimating equations (GEE) analysis was conducted to explore changes in mYPAS scores across the three time points while controlling for differences in independent variables between the treatment groups. The GEE model was used to account for the repeated measurements and non-normality of the mYPAS scores. This model used a gamma distribution with log link function and an unstructured correlation matrix. Pairwise comparisons of factors included in the model were adjusted with Bonferroni corrections.

All statistics were performed using IBM SPSS Statistics for Windows, version 28.0 (IBM Corporation, Armonk, NY). Intention-to-treat analysis was employed. Crossovers between groups were reviewed. The investigators monitored recruitment and the fidelity of the data, but no formal data monitoring committee was established.

### Role of funding source

The funder of the study had no role in study design, data collection, data analysis, data interpretation, or writing of the report.

## Results

Sixty patients were enrolled from September 21, 2021–May 12, 2022 (Fig. 1). Of the 8230 anesthesia patients that presented during the study period, there were 84 eligible and 24 ineligible. Of these, 60 were enrolled, and 30 patients were randomized to each group. Two patients in the control group were excluded because they did not complete the SSP-2 measure, which left 30 patients in the ASE group and 28 in the control group. There was no crossover between groups. There were no significant differences in baseline characteristics between groups (Table 1). Groups were also balanced regarding clinical characteristics except for median number of intraoperative pain medications received by the patient, which was significantly lower in the ASE group (1 vs. 3, p = 0.012). Although not statistically significant, 30% (9/30) of the ASE group vs. 11% (3/28) of the control group received preoperative medications (p = 0.070). There were no differences in registration time, preoperative wait time, anesthesia ready time, case length, or recovery time between the groups (Table 1).

**Table 1:** Baseline demographic and clinical characteristics of study population.

Level/statistic	All patients, n = 58	ASE (intervention) room group, n = 30	Standard (control) room group, n = 28
Sex			
Male	43 (74.1%)	21 (70.0%)	22 (78.6%)
Female	15 (25.9%)	9 (30.0%)	6 (21.4%)
Race			
White	42 (72.4%)	24 (80.0%)	18 (64.3%)
Black	13 (22.4%)	4 (13.3%)	9 (32.1%)
Other	3 (5.2%)	2 (6.7%)	1 (3.6%)
Ethnicity			
Hispanic or Latino	0 (0.0%)	0 (0.0%)	0 (0.0%)
Non Hispanic or Latino	58 (100.0%)	30 (100.0%)	28 (100.0%)
Age (years) at procedure	7.2 (2.9)	7.0 (2.8)	7.4 (3.0)
Weight (kg) at procedure	36.9 (26.6)	34.9 (23.8)	39.0 (29.6)
Height (cm) at procedure	126.2 (22.8)	123.5 (22.7)	129.1 (22.9)
ASA status at procedure			
1	1 (1.7%)	0 (0.0%)	1 (3.6%)
2	45 (77.6%)	23 (76.7%)	22 (78.6%)
3	12 (20.7%)	7 (23.3%)	5 (17.9%)
CIBA			
Smooth	54 (93.1%)	28 (93.3%)	26 (92.9%)
Moderate	3 (5.2%)	2 (6.7%)	1 (3.6%)
Unknown	1 (1.7%)	0 (0.0%)	1 (3.6%)
Pre-op: Versed	9 (15.5%)	6 (20.0%)	3 (10.7%)
Pre-op: Precedex	3 (5.2%)	3 (10.0%)	0 (0.0%)
Pre-op: Ketamine	2 (3.4%)	2 (6.7%)	0 (0.0%)
Pre-op meds			
No	46 (79.3)	21 (70.0)	25 (89.3)
Yes	12 (20.7)	9 (30.0)	3 (10.7)
Number of pre-op meds			
0	46 (79.3%)	21 (70.0%)	25 (89.3%)
1	10 (17.2%)	7 (23.3%)	3 (10.7%)
2	2 (3.4%)	2 (6.7%)	0 (0.0%)
Pre-op med route (n = 12)			
Oral	9 (75.0%)	6 (66.7%)	3 (100.0%)
Intranasal	1 (8.3%)	1 (11.1%)	0 (0.0%)
Intravenous	1 (8.3%)	1 (11.1%)	0 (0.0%)
Intramuscular	1 (8.3%)	1 (11.1%)	0 (0.0%)
Intra-op: Fentanyl	16 (27.6%)	6 (20.0%)	10 (35.7%)
Intra-op: Morphine	26 (44.8%)	13 (43.3%)	13 (46.4%)
Intra-op: Dilaudid	1 (1.7%)	0 (0.0%)	1 (3.6%)
Intra-op: Precedex	34 (58.6%)	16 (53.3%)	18 (64.3%)
Intra-op: Ketamine	4 (6.9%)	2 (6.7%)	2 (7.1%)
Intra-op: Versed	1 (1.7%)	1 (3.3%)	0 (0.0%)
Intra-op: Acetaminophen	22 (37.9%)	8 (26.7%)	14 (50.0%)
Intra-op: Ketorolac	24 (41.4%)	9 (30.0%)	15 (53.6%)
Number of intra-op pain meds^a^			
0	4 (6.9%)	2 (6.7%)	2 (7.1%)
1	17 (29.3%)	14 (46.7%)	3 (10.7%)
2	9 (15.5%)	4 (13.3%)	5 (17.9%)
3	21 (36.2%)	8 (26.7%)	13 (46.4%)
4	5 (8.6%)	1 (3.3%)	4 (14.3%)
5	2 (3.4%)	1 (3.3%)	1 (3.6%)
Registration time (min)	9 [8]	9 [8]	8 [11]
Pre-operative wait time (min)	74 [44]	73 [47]	78 [45]
Anesthesia ready time (min)	9 [5]	10 [4]	9 [7]
Case length (min)	51 [41]	44 [40]	54 [41]
Recovery length (min)	46 [34]	40 [34]	47 [39]
Number of pre-op meds	0.0 [0.0]	0.0 [1.0]	0.0 [0.0]
Number of intra-op pain meds^b^	2.0 [2.0]	1.0 [2.0]	3.0 [1.0]

Data are n (%), mean (SD), or median [IQR]. ASA = American Society of Anesthesiology. ASE = adaptive sensory environment. CIBA = Child Induction Behavioral Assessment Scale. min = minutes.

a See comparison of median number of intra-op meds analyzed in the table for baseline quantitative variables. Since the number of meds ranges from 0 to 5 and the unadjusted p value is close to 0.05, no multiple paired comparisons were made.

b p = 0.012.

No significant differences were found in mYPAS scores between the intervention and control groups at the three time periods (Table 2). We found no significant differences in the PES survey and overall experience scores between the two groups (Supplementary 5, Table 3). For the SSP-2 scales, if a patient was missing a value for a quadrant item, the average of the patient's values for that quadrant was used for the item. One patient in the ASE group was missing all values from the behavioral scale, so was excluded from all subscales that used one or more items from the behavioral scale. For the SSP-2 Survey, we observed a significantly higher sensitivity quadrant raw score in the ASE group compared to the control group (p = 0.0040). No other significant differences between groups were observed in all other quadrant raw scores (Supplementary 6, Table 4).

**Table 2:** Primary outcome – Total Modified Yale Preoperative Anxiety Scale (mYPAS) scores from the study population.

	ASE (intervention) room group n = 30	Standard (control) room group n = 28	Between group mean difference (95% CI)	p value^a^
Registration total score	43.5 (21.0)	42.0 (14.3)	1.5 (−8.0, 11.1)	0.88
Nursing Intake total score	47.8 (25.1)	48.4 (22.9)	−0.6 (−13.2, 12.1)	0.76
Transition total score	36.4 (19.0)	43.8 (20.7)	−7.4 (−17.8, 3.1)	0.15

Data are mean (SD). ASE = adaptive sensory environment. CI = confidence interval.

a Mann–Whitney U tests.

The GEE analysis explored changes in mYPAS scores across the three time points, controlling for SSP-2 sensitivity/sensor scores and pre-operative medications (no vs. yes). The preoperative medications variable was included because it differed between the groups by 19%, although the difference was not statistically significant.

In the model, group, timepoint, SSP-2 scores, preoperative medications, and the group x time point interaction were not associated with mYPAS scores. The main effect of time point was statistically significant for nursing intake vs. transition to operating room, with a decrease of 8.1 points across both groups combined (p = 0.014). Within groups, the decrease was statistically significant in the ASE group (11.4 points, p = 0.030), but the difference was less than 15 points, considered the minimum clinically important difference (MCID). The decrease in the control group was not statistically significant (4.6 points, p = 0.39).

There were no noted adverse events, withdrawals or complaints related to the study. Several families refused participation as they requested to use the ASE rooms; these families had used them previously.

Post-study power calculations indicated the study has adequate power. The study enrolled 60 patients, of which 58 (30 ASE room, 28 control room) were included in the analyses. The pooled standard deviations were 18.0 at Registration, 23.8 at Nursing Intake, and 20.0 at Transition. For a total sample size of 58, the power to detect a difference of 15 points between groups at Registration was 93%, 77% at Nursing Intake, and 88.7% at Transition. The largest difference between the groups was −7.4 points at Transition, therefore the study had adequate power.

## Discussion

We successfully conducted a randomized feasibility trial in which ASE was used to reduce perioperative anxiety in patients with ASD. Despite its preliminary nature, this study shows promising results: although there was no significant difference found in mYPAS scores between study groups, there was a significant reduction in the mYPAS scores from Nursing Intake to Transition in the ASE group. This was determined from the post hoc GEE analysis that controlled for differences in SSP-2 scores and preoperative medications. The potential value of ASE in reducing preoperative anxiety found in our study is consistent with previously published dental literature.^5^, ^6^, ^7^, ^8^, ^9^, ^10^^,^^12^^,^^13^

Families reported similar patient experiences and remarkably high satisfaction ratings regardless of group allocation. This lack of difference in ratings may be related to our standard preoperative care that includes providing developmentally appropriate play items (stickers, coloring pages, play doh, etc.) and developmentally appropriate education prior to surgery. These play and preparation interventions have been found to decrease preoperative anxiety in children undergoing surgery.^41^^,^^42^ Patients in both groups received this standard of care and individualized coping plans prior to being enrolled in the study. Implementation of individualized care plans for patients with developmental disabilities leads to improved patient experiences and family satisfaction along with decreased anxiety and distress in children with ASD.^43^^,^^44^

Several limitations may influence the interpretation of our results. This was a single institution study. However, over the past several years, extensive quality improvement efforts have been made to improve the perioperative care of patients with ASD. Routine nursing education has been provided in keeping with the known benefits in improvement of quality of care in patients with developmental disabilities.^20^ Staff at our institution are now accustomed to using information in coping plans to make sensory friendly adaptations to the environment such as dimming the lights, closing the door, etc. These are in keeping with similar provisions at other facilities and acknowledge the importance of the built environment in the care of children with ASD.^32^^,^^45^^,^^46^ These interventions have led to lower rates of premedication use: only 20.7% (12/58) received premedication in our population compared to 87.5–96% reported at other institutions.^26^^,^^47^ Lower premedication did not adversely affect induction of anesthesia, with 93.1% (54/58) being classified as smooth induction on the CIBA scale, contrasting with more recent work in which children with ASD had more difficult inductions.^47^ These findings stress the importance of capacity building when caring for this vulnerable population; this is well established in the literature and generalization of these study results must be considered in this context.

Personnel including nursing staff and anesthesia providers were not blinded to group allocation. This may have led to an unconscious bias in the number of intraoperative pain medications needed as the median number of intraoperative pain medications was significantly lower in the ASE group (1 vs. 3, p = 0.012). Blinding of group allocation is not possible as nursing and anesthesia personnel need to provide care to patients while in the preoperative area and the ASE designation is readily determined.

Patients were enrolled based on a formal diagnosis of ASD, Asperger's Syndrome, or pervasive developmental disorder not otherwise specified as listed in their electronic health record. We did not evaluate or control for a number of possible baseline confounding factors that may have influenced study results. These factors include level of autism, severity of their sensory integration, verbal ability, and baseline behavioral indices. Future studies may need to incorporate these potential confounders so that they are accounted for in the interpretation of study results. Additionally, this feasibility study had a small sample size which did not permit further subgroup analysis by sex or race, we admit this as a limitation of the study.

Finally, the mYPAS was used as the primary outcome measure for this study. While it is validated as a measure of perioperative anxiety in a neurotypical population, it has not been validated for use in children with ASD.^34^ Despite this, the limited literature evaluating perioperative anxiety in this population has employed the mYPAS and we chose this instrument for this study based on this.^26^ Future study needs to focus on the development of validated measures for this population; efforts to evaluate anxiety in youth with ASD have been published but not specifically in the perioperative setting.^48^

Future clinical use of ASEs could benefit from two specific areas of further study. First, we acknowledge that the specific components of an ASE may vary depending on the patient population and clinical setting. Further research is needed to identify the most effective components of an ASE and the optimal way to implement them in clinical practice. Second, while sensory processing disorder is a common feature of ASD, it may also be seen in patients with other behavioral or developmental disorders, including primary anxiety disorders and attention deficit hyperactivity disorder (ADHD). These patients may also experience increased perioperative anxiety and may benefit from an ASE. Further research should explore the potential benefits of an ASE in these patient populations, identify potential areas of overlap with ASD, and identify strategies to implement them in clinical practice.

Our study did not provide evidence that an ASE based on a patient's individualized coping plan is effective in decreasing preoperative anxiety in patients with ASD with high sensory sensitivities compared to patients in a control group. However, the 11-point decrease in mYPAS score from Nursing Intake to Transition in the ASE group, although less than the 15-point MCID, warrants additional study. The use of an ASE requires collaboration between healthcare professionals, patients, and their families to ensure the environments are tailored to each patient's individual needs. ASE implementation deserves future investigation to generalize and optimize its use for this vulnerable population.

## Contributors

CD drafted the report, which all authors critically reviewed, edited, and approved. SA, EH, RL, TA, and SV designed the trial and intervention content. SA, EH, RL, RMR, MH, CD, and SV were responsible for trial conduct. MH, CD, RMR, and AS were responsible for database design and management. CD and AS directly assessed and verified underlying data reported in manuscript. AS performed all statistical analyses.

## Data sharing statement

Data will be shared upon reasonable request to the corresponding author. The study protocol and statistical analysis plan are publicly available.

## Declaration of interests

Dr. Sean Antosh was awarded the Robert C. Cohn Memorial Research Grant by the Dayton Children's Hospital Foundation.

## Appendix A Supplementary data

Supplementary Materials

ASE Manuscript Table of Contents
