# Supplementary material for: Use of an adaptive sensory environment in patients with autism spectrum disorder (ASD) in the perioperative environment: a parallel, randomized controlled trial
Source: Lancet Reg Health Am. 2024 Apr 18;33:100736. doi: 10.1016/j.lana.2024.100736 (PMC11031801; doi:10.1016/j.lana.2024.100736)
Supplement: Supplementary Materials [file mmc1.docx]

**Supplementary 1: Study Protocol**

**Protocol Proposal: Use of an adaptive sensory environment in patients with Autism Spectrum Disorder (ASD) in the perioperative environment**

Principal Investigator

Sean Antosh, MD

Pediatric Anesthesiologist, Dayton Children’s Hospital

Assistant Professor of Pediatrics, Wright State University Boonshoft School of Medicine

Sub-Investigators

Tahira G. Adelekan, MD, FAAP

Developmental Pediatrician, Dayton Children’s Hospital

Assistant Professor of Pediatrics, Wright State University Boonshoft School of Medicine

Robin Lawson, RN

Clinical Nurse, Perioperative Services, Dayton Children’s Hospital

Elise Huntley, MA, CCLS

Child Life Specialist, Perioperative Services, Dayton Children’s Hospital

Katelyn Shellabarger, BA, CCLS

Child Life Specialist, Perioperative Services, Dayton Children’s Hospital

Reaundra McCullough-Roach, MS

Medical Student, Wright State University Boonshoft School of Medicine

Shobhan Vachhrajani, MD, PHD, FRCSC

Pediatric Neurosurgeon and Director of Surgical Research, Dayton Children’s Hospital

Associate Professor of Pediatrics, Wright State University Boonshoft School of Medicine

Version: May 21, 2021

Study Sponsor: Department of Pediatric Anesthesiology and Perioperative Services

Funding: DCH Robert C. Cohn Memorial Research Grant

Budget: See Attached

Single-site study: Dayton Children’s Hospital, Main Campus, Perioperative Department

**Summary of Proposal**

The prevalence of patients with a diagnosis of autism spectrum disorders (ASD) has been increasing over the last several decades, currently estimated at 1 in 68 births in the United States. At Dayton Children’s Hospital, over 400 patients a year require general anesthesia for surgeries, procedures, and imaging tests. Children with ASD usually have significant language, communication, and sensory processing issues which they cope with by defining routines and rituals in their daily lives. For these patients to receive anesthesia, they must have nothing by mouth for a specific time, which can be extremely hard on their typical routine schedules. These routine changes may lead to frustration, increased anxiety, and ultimately maladaptive behaviors which can be self-injurious.

The rationale of this study is patients with ASD experience higher rates of perioperative anxiety and are highly likely to receive a premedication, however very little is known about nonpharmaceutical interventions which may decrease anxiety. From prior research in dentistry, we know there may be value in an adaptive sensory environment to ease anxiety. Current available literature is lacking in the potential benefits of an adaptive sensory friendly environment in the perioperative environment as a non-pharmaceutical intervention to decrease anxiety.

Our goal is to enroll approximately sixty ASD patients presenting to the perioperative environment for outpatient procedures over a 12 month period. The objective of this study is to evaluate the use of adaptive sensory environments to reduce ASD patients’ anxiety during the perioperative process. Additionally, we aim to obtain survey feedback from parents regarding their experiences and satisfaction throughout the course of their care. We hypothesize that an adaptive sensory environment, tailored by an individualized coping plan, will decrease preoperative anxiety in child with ASD. We believe these environments will be most important in patients with higher degrees of sensory integration severity, as determined by the Short Sensory Profile 2 (SSP-2). We also hypothesize that families will have better patient experiences and higher satisfaction ratings when an adaptive sensory environment is utilized. The long-term goal of our research is to define sensory adaptive environments in the perioperative space as an equal or superior alternative to premedication for ASD patients.

**Objectives and Specific Aims**

1. Prospectively examine the preoperative anxiety scores of ASD patients in an adaptive sensory environment.

2. Determine the relationship of severity of sensory integration in ASD patients and their preoperative anxiety scores.

3. Explore family satisfaction with tailored care of their ASD child in the peri-operative environment.

**Background Information and Previous Studies**

As the prevalence of autism spectrum disorders continues to increase, the need for sedation and/or general anesthesia for various procedures will continue to increase as well. In the last year at Dayton Children’s, we have seen over 400 patients for surgical procedures, imaging studies, and other non-invasive procedures. Patients with ASD have set routines that are interrupted by surgery and anesthesia. For example, they may not be able to eat or drink anything after a certain time or need to change their clothes into a hospital gown. These small changes may induce maladaptive behaviors which could include self-harm. Children with ASD are known to experience higher rate of perioperative anxiety1, which may require pharmaceutical intervention. Typically, the premedication type and route of administration is different in patients with ASD compared to those with who are neurotypical.2 Often in these scenarios, it is an intramuscular injection of ketamine which may require physical restraint of the patient by their caretakers and staff leading to a potential safety risk.

Adverse events are more likely to occur if failure to consider child’s routines, special interest, sensory sensitivities, and level of understanding.3 Current practice at Dayton Children’s Hospital is to obtain an individualize coping plan by our child life specialists prior to the morning of anesthesia. Coping plans have been shown to be helpful in the perioperative management of children with ASD and determining the need for preoperative sedation based on severity levels.4 There is emerging evidence from dental literature that a sensory friendly environment can help relax ASD patients during routine procedure in the office.5 Thus far, there is no research that has expanded the knowledge into a hospital and medical setting, particularly the perioperative environment.

**Methodology**

At Dayton Children’s, all patients with autism or other developmental delays are called prior to the day of surgery by child life specialists to obtain a coping plan. The coping plan is a document that details the patient’s previous medical experiences and provides information regarding their communication abilities, specific interests, sensory sensitivities, and triggers that make them upset. Additionally, the child life specialists elicit information regarding the best environment for the child and if they have any comfort items. The above information is documented into the EHR for all staff members to see prior to taking care of the patient the day of surgery.

For this study, inclusion criteria will include patients with a formal diagnosis of Autism Spectrum Disorder, Asperger’s Syndrome, or pervasive developmental disorder NOS patients, who are aged three to twelve years old, presenting for outpatient surgery at main campus. Patients will be excluded if a coping plan is not able to be obtained prior to day of surgery, have an American Society of Anesthesiology (ASA) risk score greater than 3, non-English speaking, or refusal of participation by guardian. Those patients that meet the criteria will be consecutively approached via telephone call 24-48 hours prior to procedure by the research team to describe the study and gauge interest in participating until we recruit sixty patients.

The study will recruit a total of sixty patients in two parallel groups randomized to 1:1 allocation: control group (standard practice with no sensory adaptive environment) or intervention group (sensory adaptive environment). Patients will be randomized in varying block sizes using a random number generator to ensure equal numbers in each group. The random allocation, block sizes, and block sequences will be concealed from study personnel and each patient until the time a patient is assigned to a group.

After registration and informed consent, the patient will be placed in either a standard preoperative room (control) or the dedicated sensory rooms (intervention) in the preoperative surgery area based on prior randomization. For the standard practice environment group, the patient will be reserved a standard room in the preoperative area of main campus. This room will not include any additional sensory equipment as described below. The child will be allowed to use any comfort items the family brought with them or offered a hospital iPad, as is current practice for all outpatient surgery patients. For the sensory adaptive environment group, one of the three dedicated adaptive sensory rooms in the preoperative area of main campus will be set up by nursing and child life staff in accordance with the patient’s coping plan and individual needs regarding sound, light, activity level, and other stimuli. The equipment may include a portable popcorn tube with fiberoptic cart, handheld marble panel, color changing floor tiles, other sensory friendly objects, and individual sensory toys. This room will be set up prior to the patient’s arrival the day of surgery and reserved for their use. If a family requests a change from the study randomized perioperative environment, their request will be granted as to not cause potential anxiety or adverse harm to the patient.

The patient’s behaviors will be recorded by a research personnel at three time points during the preoperative waiting time. These time points will be at registration in the surgery lobby, preoperative nurse intake in the individual room, and immediately prior to transition to operating room. The validated modified Yale Preoperative Anxiety Scale (mYPAS) will be utilized to assess anxiety and behaviors. The mYPAS is the most widely used tool for assessing preoperative anxiety in children. It has high reliability, concurrent validity, and construct validity in children aged 2-12 years.6 The mYPAS consists of 5 items; 4 of the items (Activity, Emotional Expressivity, State of Apparent Arousal, and Use of Parent) are rated on scales of 1-4, and 1 item (Vocalizations) is rated on a scale of 1-6. The total score is determined by dividing each item rating by its highest possible rating, summing the results, dividing by 5, and then multiplying by 100. Total scores can range from 23.33 to 100, with higher scores representing greater anxiety. The total score on the mYPAS will be the primary outcome for the study.

During the induction of anesthesia by the assigned anesthesia personnel, the patient will be evaluated in the operating room utilizing the validated Child Induction Behavioral Assessment Scale (CIBA). The CIBA is a 3-category behavioral assessment for documenting children’s behavioral responses to induction of anesthesia. The 3 categories, Smooth, Moderate, and Difficult have associated behavioral descriptions that are scored at the time of anesthesia induction. The reliability and concurrent validity with the Induction Compliance Checklist were shown to be high in a 2020 study of children aged 1-12 years.7 As is current practice for all patients, the CIBA will be evaluated by the assigned anesthesia staff and documented in the EHR.

While the patient is in the procedure, families will fill out two questionnaires in the waiting room. The first is a 23-item questionnaire (Patient Experience Study Survey) regarding the patient preoperative experience. The first section includes yes/no questions regarding parent’s discussions with Dayton Children’s Child Life prior to the day of surgery in developing the coping plan. The second and third sections use positively worded statements measured on 5-point Likert scales from 1 = strongly disagree to 5 = strongly agree. These two sections discuss the preoperative holding room and environment on the day of surgery (9 items), and how the perioperative team related to the patient (5 items). Total scores across all items will be determined, as well as scores within each of the 3 sections. A final item will ask the family to rate the overall experience on a 10-point scale from 1 = very dissatisfied to 10 = very satisfied.

The second questionnaire is the Short Sensory Profile 2 (SSP-2), which assesses the patient’s sensory processing abilities. The SSP-2 score will be used as a surrogate for the severity of sensory sensitivity in the study patient. The SSP-2 consists of 34 behavioral items measured on 5-point Likert scales from 1 = almost never to 5 = almost always. Items are divided into 4 quadrants for scoring, Seeking (7 items), Avoiding (9 items), Sensitivity (10 items), and Registration (8 items) based on Dunn’s Sensory Processing Framework.7 Two subscale scores, Sensory Processing and Behavioral Responses Associated with Sensory Processing, as well as total scores are also determined. Raw scores are compared to a normal curve and Sensory Profile 2 Classification System developed from a normative sample. Scores >2 standard deviations (SD) below the mean are classified as “much less than others”, those between 1 and 2 SD below the mean are “less than others”, scores between 1 and 2 SD above the mean are “more than others”, and >2 SD above the mean are “much more than others”.8,9 The SSP-2 will be used as an independent variable to compare baseline responses to sensory stimuli between the control and intervention groups. If the groups differ, the SSP-2 scores will be included as a covariate in statistical analyses.

In addition to the SSP-2, the following independent variables will be extracted from the medical record: patient demographics (sex, age, weight), ASA score designated by anesthesiologist, diagnosis and health history, procedure, home medications, preoperative medications including dose and route, intraoperative medications, event times and durations (including preoperative wait time, transport to OR time, induction to anesthesia ready, case length, & recovery time), and first recovery room pain score (documented by PACU RN via FLACC score).

All data will be identified by a code number and will be transferred to a password-protected database for storage and analysis by the research investigator or assistant. All paper documentation will be secured in a locked cabinet until the conclusion of the study, at which time it will be properly disposed.

**Data Analysis**

Descriptive statistics: all continuous outcomes and independent variables will be assessed for normality and summarized with mean (SD) and range if normally distributed, or median (interquartile range, IQR) if the distribution is skewed. Categorical variables will be summarized with frequencies and percentage of non-missing values.

Comparisons between the control and intervention groups: initial statistical analyses will consist of univariate tests comparing all outcomes and independent variables between the control and intervention groups. Normally distributed continuous variables will be compared with two-sample t tests, and skewed continuous variables with Wilcoxon rank sums tests. Categorical variables will be compared between groups with chi-square tests, or Fisher’s exact tests if 1 or more expected cell frequencies is less than 5. For the mYPAS, comparisons between groups will be made at each of the 3 time periods. Differences within groups between the mYPAS scores at the 3 time periods will be determined with one-way repeated measures analysis of variance (ANOVA) or Friedman’s ANOVA, depending on the distribution of the data.

If randomization does not result in the groups being balanced for any of the independent variables, particularly the SSP-2, multivariable statistical analyses will be conducted to control for the variables that are different between the groups. For mYPAS scores and scores on the Patient Experience Survey, either multiple linear regression or generalized linear models will be used, depending on the distribution of the outcomes. For the CIBA, with 3 levels of responses, ordinal logistic regression will be used to compare the response levels between groups, controlling for any confounding variables.

An intention-to-treat analysis, in which participants will be analyzed based on the group they were initially allocated to, will be employed. Degree of crossover, in which families request placement in the opposite group from allocation, will be reviewed. Should there be significant crossover of participants, then data will also be analyzed according to the groups in which patients were managed.

Sample size estimation: in previous randomized clinical trials using the mYPAS, investigators considered a difference between groups of ≥15 points to be clinically meaningful.10-12 Standard deviations reported in 1 of the studies ranged from 4.2-18.1.12 Using alpha = 0.05, power = 0.80, and pooled SD = 18.1, 23 patients would be required in each group to detect a difference of 15 points in mean mYPAS scores. Allowing for a 10% drop out rate, 26 patients in each group or 52 patients in total would be required.

Departments involved/supporting: Department of Pediatric Anesthesiology, Child Life and Perioperative Services

**Data collection**

What data will be collected?: Patient anxiety scores by utilizing the validated modified Yale Preoperative Anxiety Scale (mYPAS), Child Induction Behavior Assessment Scale (CIBA) by the assigned anesthesia staff and documented in the EHR, Short Sensory Profile 2 and Patient Experience Survey will be documented by parent. The following independent variables will be extracted from the medical record: patient demographics (sex, age, weight), ASA score designated by anesthesiologist, diagnosis and health history, procedure, home medications, preoperative medications including dose and route, intraoperative medications, event times and durations (including preoperative wait time, transport to OR time, induction to anesthesia ready, case length, & recovery time), and first recovery room pain score (documented by PACU RN via FLACC score).

Who will collect and enter the data?: Robin Lawson, clinical nurse, will collect the anxiety scores and parent questionaires. Reaundra McCullough-Roach,MS will collect the remainder of the data from the EHR and enter in to study spreadsheets.

What data collection software will be used?: The data collected will be transposed into a password protected excel sheet that will be stored in a secure folder on the share drive, with limited access to only the research group.

Has the sponsor provided a Data Use Agreement?: n/a

Where will the data be stored?: The data will be stored in a secure folder on the share drive, with limited access to the research group. Paper documents will be stored in a locked filing cabinet until the termination of the study.

**Significance**

Our study aims to show that an adaptive sensory environment tailored by an individual coping plan will lead to decreased preoperative anxiety scores. We suspect it will be more beneficial in patients with higher severity of sensory sensitivities, as determined by SSP-2. Additionally, we aim to show families have better patient experiences and increased satisfaction with their care.

The long-term goal of our research is to define sensory adaptive environments in the perioperative space as an equal or superior alternative to premedication for ASD patients. We foresee extension of these environments into the anesthesia induction process, as well as the postoperative care in the recovery room. Ultimately, sensory adaptive environments may be beneficial in all realms of healthcare experience, including but not limited to outpatient, inpatient, urgent care, and emergency locations. From this study, we hope to gain data and insight into the utility of sensory environments that may be broadened to these environments.

**Risk factors to patients**

Since this study does not require any additional tests, procedures, or treatments, there are no known physical risks from participation in this study. There may be a slight risk of mental discomfort of the patient’s guardian while completing the forms. There is a small risk of unauthorized release of information. All efforts will be made to maintain confidentiality.

**Protection of Privacy**

All participants will obtain a series number. All data collected will be identified by the participants series number. All identifying information will be kept separate and protected from the data collected.

**Assent and permission**

Written informed consent will be obtained according to institutional protocol on the day of surgery during the registration process. Assent will not be obtained from any participants due to their limited baseline cognitive function and abilities. Please see attached proposed informed consent form.

No information acquired will be used as a part of a diagnostic or treatment program.

**References**

1) Elliott A, Holley A, Ross A, Soleta A, Koh J. A prospective study comparing perioperative anxiety and posthospital behavior in children with autism spectrum disorder vs typically developing children undergoing outpatient surgery. Pediatr Anesth. 2018; 28:142–48.

2) Arnold B, Elliott A, Laohamroonvorapongse D, Hanna J, Norvell D, Koh, J. Autistic children and anesthesia: is their perioperative experience different? Paediatr Anaesth, 2015; 25:1103-10.

3) Taghizadeh N, Davidson A, Williams K, Story D. Autism spectrum disorder (ASD) and its perioperative management. Paediatr Anaesth. 2015; 25:1076-84.

4) Swartz J, Amos K, Brindas M, Girling L, Ruth Graham M. Benefits of an individualized perioperative plan for children with autism spectrum disorder. Pediatr Anesth. 2017; 27: 856– 62.

5) Cermak S, Stein Duker L, Williams M, Dawson M, Lane C, Polido J. Sensory Adapted Dental Environments to Enhance Oral Care for Children with Autism Spectrum Disorders: A Randomized Controlled Pilot Study. J Autism Dev Disord. 2015; 45:2876-88.

6) Kain Z, Mayes L, Cicchetti D, Bagnall A, Finley J, Hofstadter M. The Yale Preoperative Anxiety Scale: how does it compare with a "gold standard"? Anesth Analg. 1997; 85: 783-88.

7) Winterberg A, Ding L, Hill L, Stubbeman B, Varughese A. Validation of a simple tool for electronic documentation of behavioral responses to anesthesia induction. Anesth Analg. 2020; 130:472-79.

8) Simpson K, Adams D, Alston-Knox C, Heussler H, Keen D. Exploring the sensory profiles of children on the autism spectrum using the Short Sensory Profile-2 (SSP-2). J Autism Dev Disord. 2019; 49: 2069-79.

9) Chojnicka I, Pisula E. Adaptation and psychometric properties of the Polish version of the Short Sensory Profile 2. Medicine (Baltimore). 2019; 98:e17689.

10) Hanna A, Ramsingh D, Sullivan-Lewis W et al. A comparison of midazolam and zolpidem as oral premedication in children, a prospective randomized double-blinded clinical trial. Paediatr Anaesth. 2018; 28: 1109-15.

11) Kerimoglu B, Neuman A, Paul J, Stefanov D, Twersky R. Anesthesia induction using video glasses as a distraction tool for the management of preoperative anxiety in children. Anesth Analg. 2013; 117: 1373-79.

12) Lee J, Lee J, Lim H et al. Cartoon distraction alleviates anxiety in children during induction of anesthesia. Anesth Analg. 2012; 115: 1168-73.

**Supplementary 2: mYPAS**

**Supplementary 3: PES Survey**

**Supplementary 4: SSP-2**


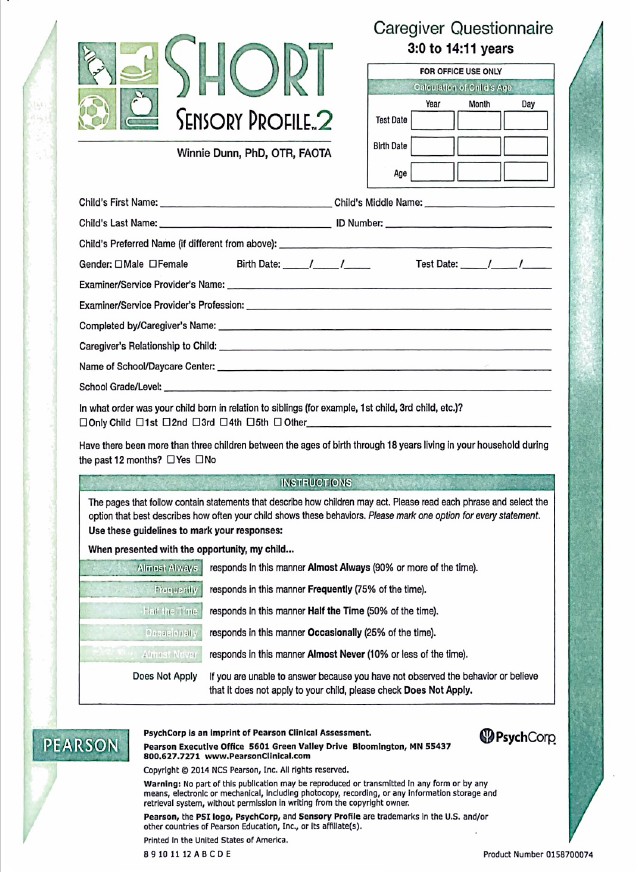


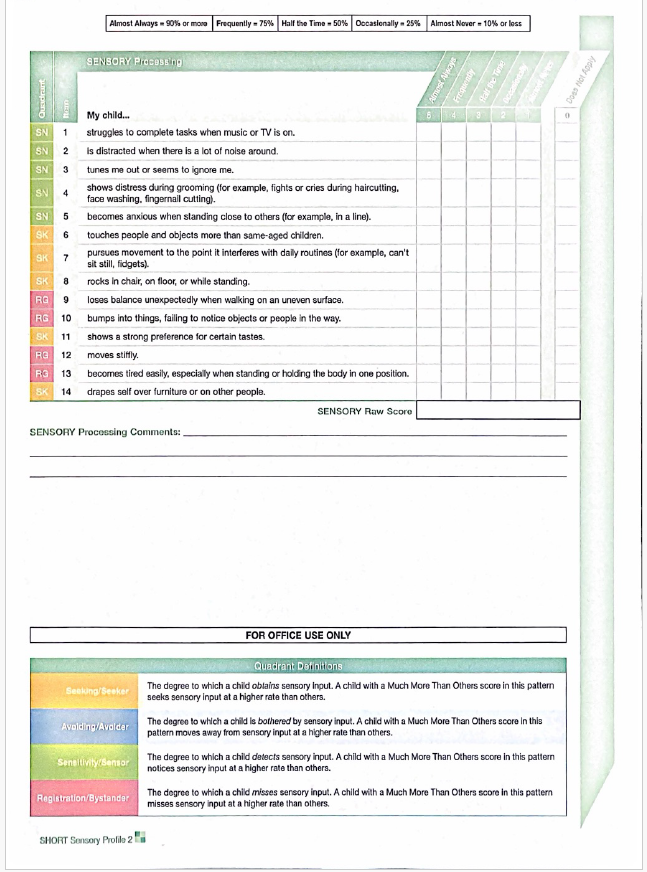


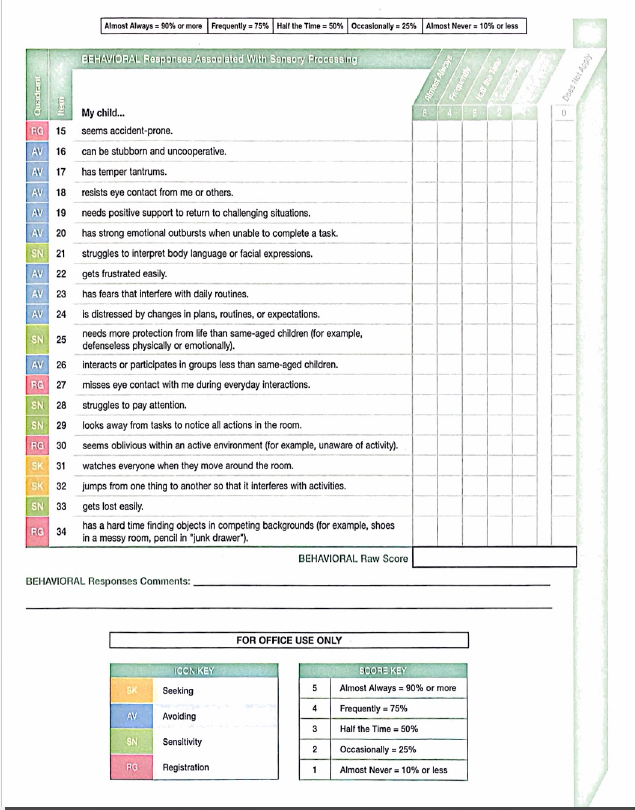


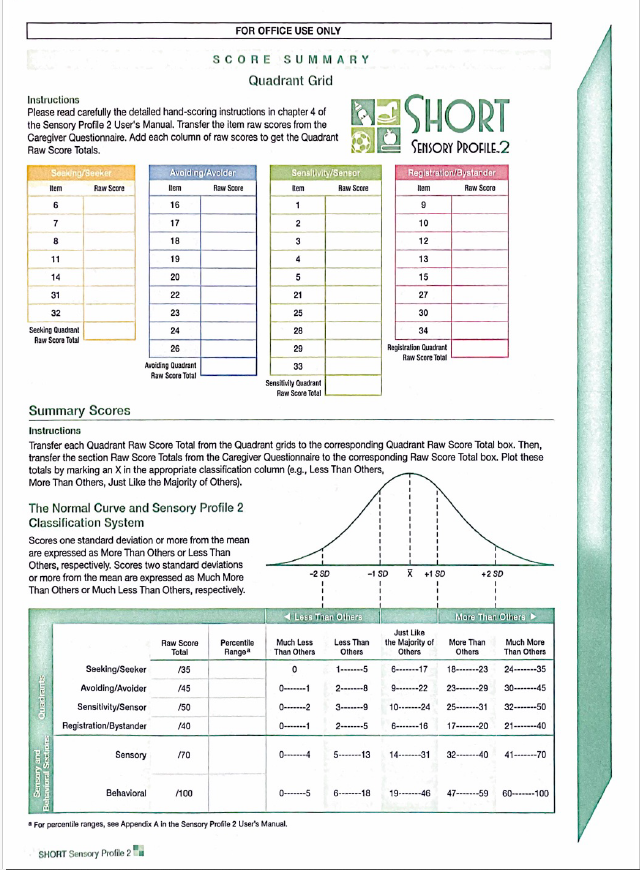


**Supplementary 5: Table 3**

**Supplementary 6: Table 4**
