# Supplementary material for: Use of an adaptive sensory environment in patients with autism spectrum disorder (ASD) in the perioperative environment: a parallel, randomized controlled trial
Source: Lancet Reg Health Am. 2024 Apr 18;33:100736. doi: 10.1016/j.lana.2024.100736 (PMC11031801; doi:10.1016/j.lana.2024.100736)
Supplement: ASE Manuscript Table of Contents [file mmc2.docx]

**Use of an Adaptive Sensory Environment in Patients with Autism Spectrum Disorder (ASD) in the Perioperative Environment: A Parallel, Randomized Controlled Trial**

**Table of Contents**

Title Page

Abstract 1

Manuscript 1-8

Table 1: Baseline demographic and clinical characteristics 9

Table 2: Primary Outcome - mYPAS scores 10

Figure 1: Trial Profile 11

Figure 2: ASE room vs. Control room images 12-13

Completed CONSORT RCT checklist 14-16

Research in Context Panel 17

Supplementary materials

Supplementary 1: ASE Study Protocol 1-7

Supplementary 2: Validated mYPAS 8

Supplementary 3: Patient Experience Study (PES) Survey 9

Supplementary 4: Short Sensory Profile 2 (SSP-2) Questionnaire 10-13

Supplementary 5: Table 3: Secondary Outcome - PES Survey Scores 14

Supplementary 6: Table 4: Secondary Outcome - SSP-2 scores 15
